# PacBio But Not Illumina Technology Can Achieve Fast, Accurate and Complete Closure of the High GC, Complex *Burkholderia pseudomallei* Two-Chromosome Genome

**DOI:** 10.3389/fmicb.2017.01448

**Published:** 2017-08-02

**Authors:** Jade L. L. Teng, Man Lung Yeung, Elaine Chan, Lilong Jia, Chi Ho Lin, Yi Huang, Herman Tse, Samson S. Y. Wong, Pak Chung Sham, Susanna K. P. Lau, Patrick C. Y. Woo

**Affiliations:** ^1^Department of Microbiology, Li Ka Shing Faculty of Medicine, The University of Hong Kong Hong Kong, Hong Kong; ^2^State Key Laboratory of Emerging Infectious Diseases, Department of Microbiology The University of Hong Kong, Hong Kong, Hong Kong; ^3^Research Centre of Infection and Immunology The University of Hong Kong, Hong Kong, Hong Kong; ^4^Carol Yu Centre for Infection, The University of Hong Kong Hong Kong, Hong Kong; ^5^Centre for Genomic Sciences, The University of Hong Kong Hong Kong, Hong Kong; ^6^Department of Psychiatry, The University of Hong Kong Hong Kong, Hong Kong; ^7^Collaborative Innovation Center for Diagnosis and Treatment of Infectious Diseases, The University of Hong Kong Hong Kong, Hong Kong

**Keywords:** complete, genome, PacBio RS II, P6-C4, *Burkholderia pseudomallei*

## Abstract

Although PacBio third-generation sequencers have improved the read lengths of genome sequencing which facilitates the assembly of complete genomes, no study has reported success in using PacBio data alone to completely sequence a two-chromosome bacterial genome from a single library in a single run. Previous studies using earlier versions of sequencing chemistries have at most been able to finish bacterial genomes containing only one chromosome with *de novo* assembly. In this study, we compared the robustness of PacBio RS II, using one SMRT cell and the latest P6-C4 chemistry, with Illumina HiSeq 1500 in sequencing the genome of *Burkholderia pseudomallei*, a bacterium which contains two large circular chromosomes, very high G+C content of 68–69%, highly repetitive regions and substantial genomic diversity, and represents one of the largest and most complex bacterial genomes sequenced, using a reference genome generated by hybrid assembly using PacBio and Illumina datasets with subsequent manual validation. Results showed that PacBio data with *de novo* assembly, but not Illumina, was able to completely sequence the *B. pseudomallei* genome without any gaps or mis-assemblies. The two large contigs of the PacBio assembly aligned unambiguously to the reference genome, sharing >99.9% nucleotide identities. Conversely, Illumina data assembled using three different assemblers resulted in fragmented assemblies (201–366 contigs), sharing only 92.2–100% and 92.0–100% nucleotide identities to chromosomes I and II reference sequences, respectively, with no indication that the *B. pseudomallei* genome consisted of two chromosomes with four copies of ribosomal operons. Among all assemblies, the PacBio assembly recovered the highest number of core and virulence proteins, and housekeeping genes based on whole-genome multilocus sequence typing (wgMLST). Most notably, assembly solely based on PacBio outperformed even hybrid assembly using both PacBio and Illumina datasets. Hybrid approach generated only 74 contigs, while the PacBio data alone with *de novo* assembly achieved complete closure of the two-chromosome *B. pseudomallei* genome without additional costly bench work and further sequencing. PacBio RS II using P6-C4 chemistry is highly robust and cost-effective and should be the platform of choice in sequencing bacterial genomes, particularly for those that are well-known to be difficult-to-sequence.

## Introduction

Since the release of the first complete bacterial genome sequence in 1995 (Fleischmann et al., 1995), genome sequencing has been the cornerstone of studying any bacterial species. In the 1990s and early 2000s, bacterial genome sequencing was performed by the random shotgun approach, through physical shearing of the bacterial chromosomal DNA, cloning of the sheared fragments, sequencing individual clones and assembling the sequences using computer software. However, this approach using low-throughput long-read Sanger sequencing is extremely labor intensive and expensive. In the last decade, DNA sequencing technology has undergone a breakthrough from the traditional Sanger sequencing to a number of high-throughput short-read second generation sequencing technologies, which began with the release of the 454 pyrosequencing platform in 2005 (Margulies et al., 2005), but it has subsequently been dominated by the Illumina platforms with the HiSeq instrument being the most popular one. The Illumina HiSeq platform utilizes sequencing by synthesis technology where fluorescently labeled reversible terminator nucleotides are incorporated into growing DNA strands and imaged via their fluorophore excitation at the point of incorporation. This method provides true base-by-base sequencing that virtually eliminates errors and up to 750 Gb of data can be produced per sequencing run. Accordingly, this platform is the industry standard in terms of accuracy and throughput in second generation sequencing. Despite these advantages, Illumina platforms are limited by its read length, currently ranging from 25 to 300 bases, and as it requires PCR amplification of multiple DNA templates before sequencing, there is potential for base-composition bias which may bias the G+C content of the sequences (Goodwin et al., 2016).

In 2011, Pacific Biosciences introduced the first PacBio RS sequencing platform (using first generation chemistry, P1-C1) to the market. This machine uses single molecule real-time (SMRT) detection technology that achieves real-time sequencing of individual polymerase molecules (Eid et al., 2009). SMRT detection is based on the properties of zero-mode waveguides (ZMWs), consisting of DNA polymerases bound to nanophotonic confinement structures, and DNA is synthesized from a sample template within an array of ZMWs using fluorescent-labeled nucleotides. The ZMWs create an illuminated observation volume and the fluorophore excitation of a single nucleotide incorporated into the growing DNA strand is detected. Simultaneous detection of individual fluorophores in real-time is then generated for each ZMW while the DNA strand is synthesized. This technology does not require amplification of the genomic DNA, which addresses one of the major problems of second generation sequencing technologies; thus leading to the least degree of bias and longer read lengths (∼2,500 to 23,000 bp). However, this technology is limited by its tendency to have error. In fact, errors have been reported in more than 10% of the reads, mainly indel events, which may be due to the short time intervals between nucleotide incorporation events that prevent the fluorophore excitation from being correctly detected. Nevertheless, these errors are rather random and can be detected and algorithmically managed. With improvement of its chemistry and software, a new version of the sequencer, using the latest P6-C4 chemistry, has markedly increased accuracy, throughput and read length compared to earlier models (Rhoads and Au, 2015).

Despite all these advantages of the PacBio RS sequencing platforms and their potential problems, no study has directly compared the new version of sequencer, PacBio RS II that uses the latest P6-C4 chemistry, with the second generation sequencing Illumina HiSeq 1500 platform for sequencing of the same bacterium. In this study, we evaluated and compared the robustness of these two platforms in sequencing a strain of *Burkholderia pseudomallei* recovered from a patient with fatal disseminated melioidosis. *B. pseudomallei* infections are endemic in Southeast Asia and emerging in the Western world. *B. pseudomallei* was chosen for this study because of the complexity of its genome which is composed of two large circular chromosomes with a very high G+C content of 68–69%, highly repetitive regions and substantial genomic diversity, and represents one of the largest and most complex bacterial genomes sequenced.

## Materials and Methods

### Strain

The *B. pseudomallei* strain 14M0960418 (BC334) was isolated from the blood culture of an 82-year-old Chinese man in Hong Kong who had disseminated melioidosis with infected pseudoaneurysm. The patient succumbed because of massive hemoptysis due to ruptured pseudoaneurysm.

### Genomic DNA Preparation

Genomic DNA of *B. pseudomallei* strain 14M0960418 was extracted from overnight cultures grown at 37°C on blood agar using a genomic DNA purification kit (QIAgen, Hilden, Germany) according to the manufacturer’s instructions. The isolated DNA was sequenced by the PacBio RS II system (Pacific Biosciences Inc.) and Illumina HiSeq 1500 system.

### SMRTbell Library Preparation and PacBio Sequencing

Sequencing was performed by the Macrogen Inc. (Geumcheon-gu, Seoul, South Korea) with a PacBio RS II sequencer using one SMRT cell and P6-C4 chemistry with a PacBio RS II sequencer at 120 min movie length (Pacific Biosciences, Menlo Park, CA, United States). A 20-kb SMRTbell library was generated from sheared genomic DNA via a 20-kb template library preparation workflow using protocols and reagents according to the manufacturers’ instructions.

### Illumina Library Preparation and HiSeq Sequencing

Sequencing was performed by Centre for Genomic Sciences, The University of Hong Kong (HKU), using Illumina HiSeq 1500 system (Run type: PE151 bp). All sequencing operations were performed using the protocols and reagents according to our previous publications (Teng et al., 2014, 2016). The genomic DNA library was prepared using Nextera XT DNA Sample Prep Kit (Illumina, San Diego, CA, United States). Briefly, 1 ng of input DNA was tagmented by the Nextera XT transposome at 55°C for 5 min, followed by end-repair, A-tailing, adaptor ligation, and library amplification according to the manufacturer’s protocol. The DNA library was validated by Agilent Bioanalyzer (Agilent Technologies, Santa Clara, CA, United States) and Qubit system for quality control analysis. The library was denatured and diluted to optimal concentration and applied in the cluster generation steps. HiSeq PE Rapid Cluster Kit v2 (Illumina, San Diego, CA, United States) was used for cluster generation on the flow cell. Illumina HiSeq Rapid SBS Kit v2 (Illumina, San Diego, CA, United States) was used for PE151 paired-end sequencing with mean library size of 350 bp. Image analysis and base calling were performed with SCS2.8/RTA1.8 (Illumina, San Diego, CA, United States). FASTQ file generation and the removal of failed reads were performed using CASAVA ver.1.8.2 (Illumina, San Diego, CA, United States).

### Genome Assembly

Single molecule real-time sequencing reads were *de novo* assembled using the Hierarchical Genome Assembly Process (HGAP) workflow (Chin et al., 2013) in the PacBio’s open-source SMRT Analysis software suite 2.3 (Pacific Biosciences Inc., Menlo Park, CA, United States). To allow fair comparison between sequencing data generated from PacBio RS II and Illumina HiSeq platforms, three different commonly used assemblers, MIRA (Chevreux et al., 1999), SPAdes (Bankevich et al., 2012), and Velvet (Zerbino and Birney, 2008), were used to assemble the Illumina HiSeq reads. Illumina reads were first cleaned by PRINSEQ-lite 0.20.4 (Schmieder and Edwards, 2011) to remove exact identical duplicates and to trim the reads with quality scores lower than 30. Adaptor was trimmed by trim_galore 0.4.0^1^. Cleaned and adaptor free reads were then assembled by MIRA 4.9.5.2 (70× coverage), SPAdes 3.6.1 (143× coverage) and Velvet 1.2.10 (143× coverage) respectively (Chevreux et al., 1999; Zerbino and Birney, 2008; Bankevich et al., 2012). For MIRA assembly, “genome, *de novo*, accurate” parameters was used. For SPAdes and Velvet assemblies, multiple *k*-mers were tested, in which 127 and 99 *k*-mers for SPAdes and Velvet, respectively, produced the best results and was chosen for final assembly. *De novo* hybrid assembly using both PacBio subreads and trimmed Illumina reads was also performed using SPAdes with optimized *k*-mer size of 127.

### Genome Annotation and Bioinformatics Analyses

Genome annotation of each assembly was performed automatically via Rapid Annotations using Subsystems Technology (RAST) server version 2.0 (Overbeek et al., 2014). tRNAs were predicted with tRNAscan-SE (Lowe and Eddy, 1997). Ribosomal RNAs (rRNAs) were predicted with RNAmmer (Lagesen et al., 2007). Repeats were determined by Tandem Repeats Finder version 4.07b (Benson, 1999). Sequence alignments and ordering was performed using Mauve 2.3.1 using default parameters (Darling et al., 2007). Mauve identified and aligned regions of local collinearity which known as locally collinear blocks. Locally collinear blocks of the same color indicated homologous regions, and do not contain any rearrangements of homologous sequence. Geneious R8 was used as a graphical visualization tool to aid assembling and joining of contig sequence (Kearse et al., 2012).

Indicated coverage of sequencing reads from different assemblies was mapped to the reference genome sequence by BBMap using default algorithm^2^. Protein coding sequences of 3,909 core and 135 virulence proteins were searched against PacBio and Illumina assemblies using tBLASTn. BLAST results were filtered based on sequence coverage and sequence identity using the following criteria: query sequence coverage > =80% or subject sequence coverage > =80% and sequence identity > =80%. For MLST sequences, the nucleotide sequences and BLASTn were used instead. Furthermore, whole genome multilocus sequence typing (wgMLST)-based approach was used to compare the allele profile generated by different assemblies (reference genome, PacBio assembly and the three different Illumina assemblies) (Liu et al., 2016). First, a pan-genome allele database (PGAdb) was established by submitting all assembled sequences to a web service tool^3^ using default parameters, which resulted in a total of 3,394 genes for chromosome 1 and 2,295 genes for chromosome 2 detected in all assemblies. Next, sequences in each gene (locus) with mismatched nucleotide between each other were designated as different alleles using a standardized numbering system. An allelic profile consisting of a series of numbers assigned for all loci (3,394 genes for chromosome 1 and 2,295 genes for chromosome 2) was then formed for each assembly and compared to that of the reference genome sequence.

### Circular Genome Visualization

Circular visualizations were constructed by Circos (version 0.69) software package (Krzywinski et al., 2009). Sequence of contigs from different assemblies was mapped to the reference genome sequence by BLASR using default parameters (Chaisson and Tesler, 2012).

### Sequence Data

Sequences of core proteins, virulence proteins, and seven house-keeping genes of *B. pseudomallei* were retrieved, respectively, from different databases as described below. In this study, 3,909 proteins were defined as core proteins and represented proteins that are present in all the strains of *B. pseudomallei* (*n* = 28 as of October 2015) with genomes available in Prokaryotic Genome Analysis Tool (PGAT) at http://tools.uwgenomics.org/pgat/ (Brittnacher et al., 2011) (**Table 1**). Virulence proteins were selected from the Virulence Factor Database (VFDB) at http://www.mgc.ac.cn/VFs/main.htm, which contains major virulence factors for various pathogenic bacteria (Chen et al., 2005). One hundred and thirty five proteins were defined as virulence proteins and represented proteins present in all the strains of *B. pseudomallei* (*n* = 4) with complete genomes available in the database, including *B. pseudomallei* 1106a, *B. pseudomallei* 1710b, *B. pseudomallei* 668, and *B. pseudomallei* K96243. These proteins were previously linked to the pathogenesis of *B. pseudomallei*, including those related to actin-based intracellular motility, adhesin, antiphagocytosis, invasion, and secretion systems. Sequences of the seven house-keeping genes (*ace, gltB, gmhD, lepA, lipA, narK*, and *ndh*) were downloaded from the MLST database at http://bpseudomallei.mlst.net/. These gene targets have been used for MLST of *B. pseudomallei*.

**Table 1:** Genomes used for bioinformatics analyses in this study.

Strain	Genomic elements	Number of bases	Status
*B. pseudomallei* 1026a	Contigs	7,160,336	Draft assembly
*B. pseudomallei* 1026b	Chromosome 1	4,092,668	Complete
	Chromosome 2	3,138,747	
*B. pseudomallei* 1106a	Chromosome 1	3,988,455	Complete
	Chromosome 2	3,100,794	
*B. pseudomallei* 1106b	Contigs	7,214,442	Draft assembly
*B. pseudomallei* 112	Contigs	6,934,311	Draft assembly
*B. pseudomallei* 1258a	Contigs	6,767,946	Draft assembly
*B. pseudomallei* 1258b	Contigs	7,080,338	Draft assembly
*B. pseudomallei* 14	Contigs	6,730,227	Draft assembly
*B. pseudomallei* 1655	Contigs	7,030,687	Draft assembly
*B. pseudomallei* 1710a	Chromosome 1	4,115,277	Complete
	Chromosome 2	3,171,393	
*B. pseudomallei* 1710b	Chromosome 1	4,126,292	Complete
	Chromosome 2	3,181,762	
*B. pseudomallei* 305	Contigs	7,454,077	Draft assembly
*B. pseudomallei* 354a	Contigs	7,188,691	Draft assembly
*B. pseudomallei* 354e	Contigs	7,118,369	Draft assembly
*B. pseudomallei* 406e	Contigs	7,401,189	Draft assembly
*B. pseudomallei* 576	Contigs	7,246,987	Draft assembly
*B. pseudomallei* 668	Chromosome 1	3,912,947	Complete
	Chromosome 2	3,127,456	
*B. pseudomallei* 7894	Contigs	6,997,097	Draft assembly
*B. pseudomallei* 9	Contigs	6,827,079	Draft assembly
*B. pseudomallei* 91	Contigs	6,888,055	Draft assembly
*B. pseudomallei* B7210	Contigs	6,908,769	Draft assembly
*B. pseudomallei* BCC215	Contigs	7,012,758	Draft assembly
*B. pseudomallei* DM98	Contigs	6,717,096	Draft assembly
*B. pseudomallei* K96243	Chromosome 1	4,074,542	Complete
	Chromosome 2	3,173,005	
*B. pseudomallei* NCTC 13177	Contigs	7,136,682	Draft assembly
*B. pseudomallei* Pakistan 9	Contigs	7,148,557	Draft assembly
*B. pseudomallei* Pasteur 52237	Contigs	7,348,022	Draft assembly
*B. pseudomallei* S13	Contigs	7,389,720	Draft assembly


## Results

### Formation of the *B. pseudomallei* Reference Genome Sequence Using Hybrid Assembly and Genome Analysis

**FIGURE 1:**
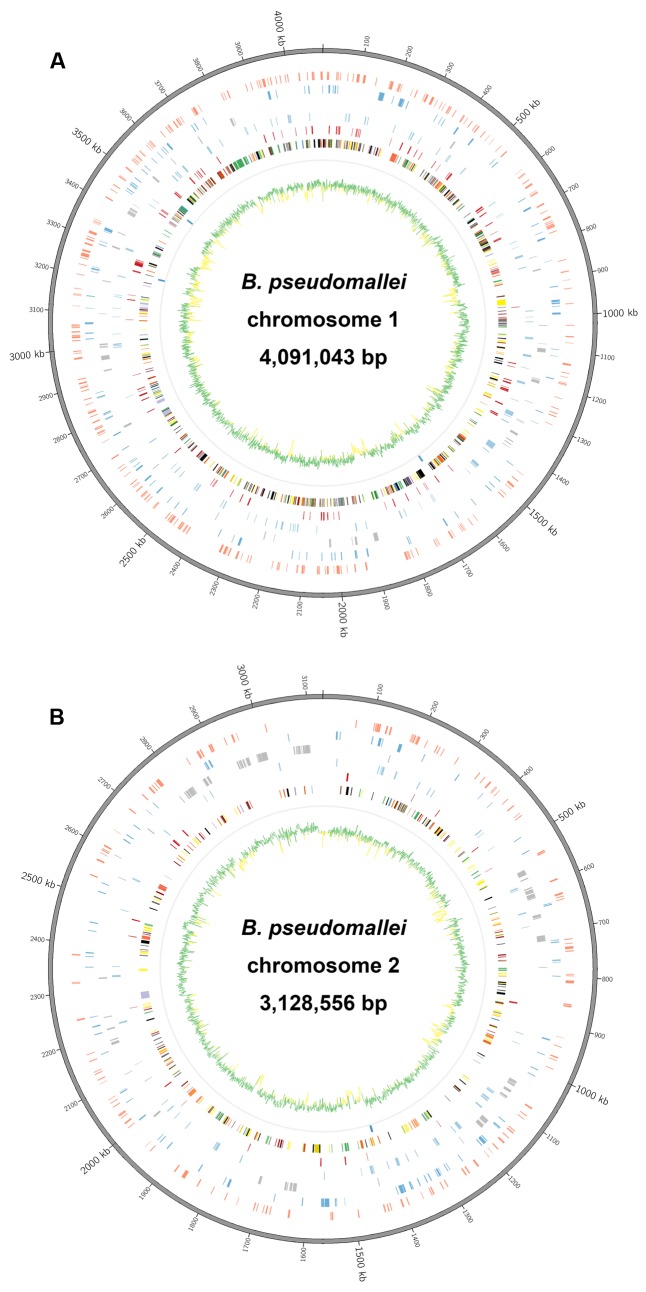
Circular representation of *Burkholderia pseudomallei* reference genome obtained by hybrid assembly. **(A)** Chromosome I and **(B)** chromosome II. CDS on each chromosome was categorized according to SEED Subsystems of Rapid Annotations using Subsystems Technology (RAST). List of tracks (from inside to outside): G+C content (green >68%, yellow <68%), ribosomal operons (dark blue), Protein Metabolism (dark red), Carbohydrates (light blue), Membrane Transport (light gray), Amino Acids and Derivatives (blue), CDSs classified into more than one category (light red), reference genome sequence (gray).

Sequence data generated from both PacBio and Illumina were used for *de novo* assembly using hybrid approach in an attempt to generate a reference genome for subsequent comparison and analyses. Such hybrid approach generated 74 contigs, ranging from 128 to 646,901 bp in length. Among these, only 24 contigs were >9,800 bp. The remaining 50 contigs were short in length (128–300 bp) with most of them being repeats. Browser-based assembling and joining of these contigs were performed manually with Geneious R8, resulting into two large contigs which were then subjected to annotation and analysis with RAST version 2.0 annotation server, Tandem Repeats Finder and RNAmmer to locate the repetitive regions. We further verified the accuracy of these two contigs by designing >100 PCR reactions flanking regions with uncertainty as well as those that contained repetitive sequences and ribosomal operons. DNA sequencing results of these >100 PCR amplicons showed that they were 100% concordant with genome sequence assembled by hybrid approach using both PacBio and Illumina data (Supplementary Table S1). These two large assembled contigs, corresponding to the chromosomes I and II (4,091,043 and 3,128,556 bp) of *B. pseudomallei*, were considered as the *B. pseudomallei* reference genome sequence for subsequent analyses and are presented in **Figures 1A,B**.

There were 7,014 protein-coding sequences (CDSs) and 71 RNAs, including 59 tRNA-encoding genes, in the reference genome according to RAST annotation. Each CDS in the annotated genome was grouped into different RAST subsystems based on the predicted functional role. Among the 7,014 CDS, only 2,791 CDSs could be categorized into RAST subsystems, representing 39.8% of total CDSs. Within the 2,791 categorized CDSs, the majority were classified into subsystems of Amino Acids and Derivatives (470 CDSs, 16.8%), Carbohydrates (354 CDSs, 12.7%), Membrane Transport (324 CDSs, 11.6%), and Protein Metabolism (266 CDSs, 9.5%). The remaining 4,223 (60.2%) CDSs could not be classified into any subsystems, with 2,084 (29.7%) of these CDSs annotated only as hypothetical proteins.

### Genome Analysis of PacBio Assembly

**FIGURE 2:**
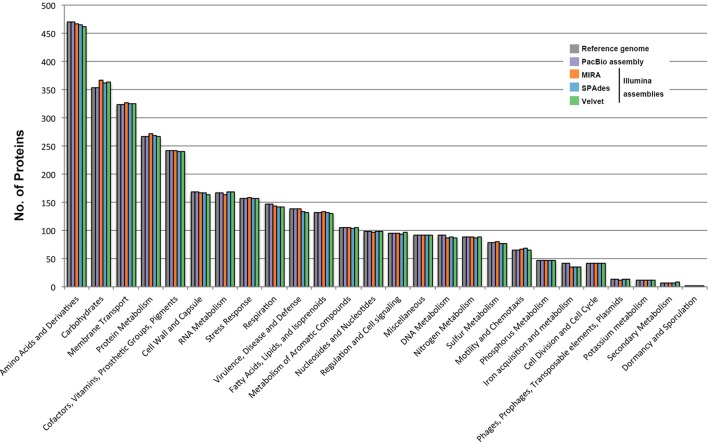
Comparison of genome sequence data of the *B. pseudomallei* strain from different assemblies. HiSeq data was assembled by three different assemblers, including MIRA, SPAdes and Velvet. Distributions of predicted coding sequence function in genomes of *B. pseudomallei* strain from different assemblies, according to SEED Subsystems of RAST are shown. The columns indicate the number of proteins in different subsystems.

The average read length of the PacBio raw data set was >8 kb with a maximum read length of about 39,000 bases. The PacBio assembly for *B. pseudomallei* strain 14M0960418 produced two large scaffolds of 4,091,945 and 3,130,290 bp in length, generating an estimated total genome size of 7,222,235 bp (Genbank accession numbers CP019042-CP019043) and was syntenic to that of the reference genome. Depth of coverage was estimated at 143× and the G+C content was 68.2%. The two scaffolds generated were submitted to RAST, resulting in 7,014 CDSs and 71 RNAs, including 59 tRNA-encoding genes (**Table 2**). The distribution of CDSs in each subsystem of PacBio assembly was also syntenic to that of the reference genome (**Figure 2**).

**Table 2:** Genome characteristics for PacBio and Illumina platforms.

Platform	PacBio RS II (latest P6-C4 chemistry)	Illumina HiSeq		
		
Assembler	SMRT analysis software suite	MIRA	SPAdes	Velvet
Total number of bases	1,000,419,819	6,278,193,333	6,278,193,333	6,278,193,333
Number of reads assembled	114,845	4,296,615^a^	7,655,760^a^	7,655,760^a^
Average depth of coverage	143×	70×^b^	143×	143×
Average read length (bp)	8,711	151	131	131
No. of contigs (>200 bp)	2	366	201	288
Largest contigs (bp)	4,091,945	152,181	372,549	299,448
Assembled genome size (bp)	7,222,235	7,261,126	7,134,451	7,137,994
N50	4,091,945	45,496	83,355	69,759
GC content (%)	68.2	68.1	68.2	68.2
Number of subsystems^c^	522	519	521	522
Number of coding sequences^c^	7,014	7,044	6,972	6,927
Number of RNAs^c^	71	73	56	60
Number of tRNA^c^	59	59	53	53


*^a^Fractions of Illumina reads were randomly selected to achieve the indicated coverage for *de novo* assembly.*

*^b^MIRA only allows *de novo* assembly with no more than 70× coverage of data.*

*^c^Annotated by RAST version 2.0.*

### Genome Analysis of Illumina Assembly

Illumina sequencing generated 27,096,399 × 2 paired-end reads with 151 bp in each read. Low quality sequences and adaptors were trimmed and generated 6,278,193,333 nucleotides (estimated 869× coverage). The Illumina data were *de novo* assembled by three different commonly used assembly programs, MIRA, SPAdes, and Velvet, which produced draft genomes ranging from 7,134,451 to 7,261,126 bp in length and distributed in 201 to 366 large contigs (>200 bp). The G+C contents ranged from 68.1 to 68.2%. Contigs generated by each assembly program were submitted to RAST, respectively, resulting in 6,927 to 7,044 CDSs and 56 to 73 RNAs, including 53 to 59 tRNA-encoding genes (**Table 2**). Among the 6,927 to 7,044 CDS generated by these three draft genomes, only 2,768 to 2,790 CDSs could be categorized into RAST subsystems, representing 39.6 to 40.0% of total CDSs. When we compared the distribution of CDSs in each subsystem of these three draft genomes with that of the reference genome, all of them had a similar percentage of their genome annotated to each subsystem (**Figure 2**).

### Characteristics of Sequence Read

**FIGURE 3:**
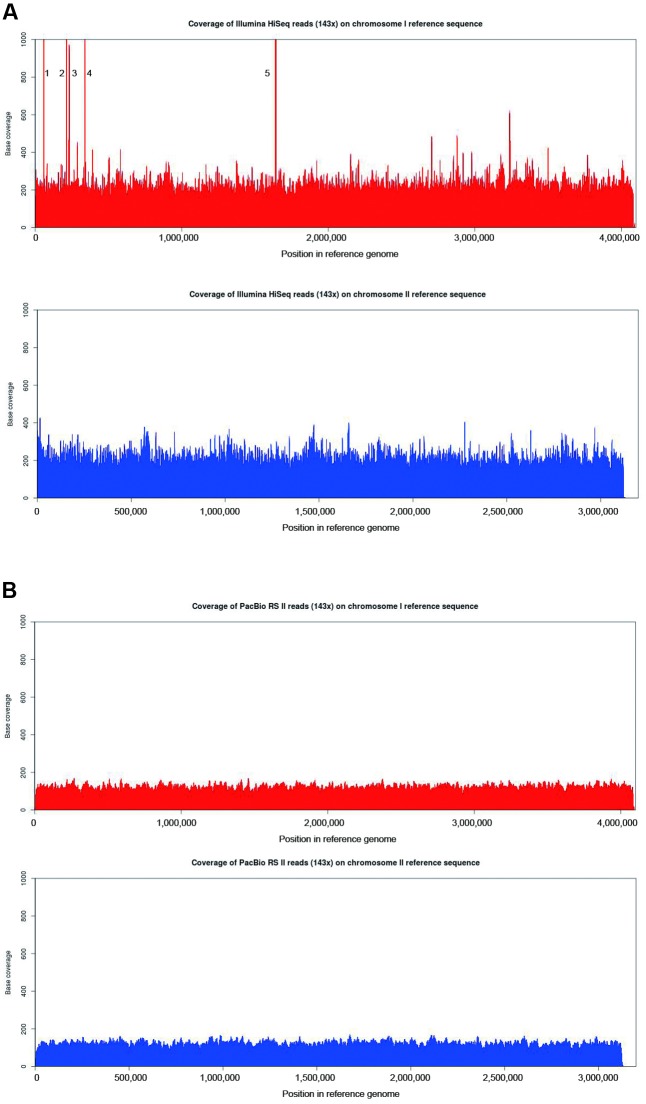
Comparison of base-per-base coverage of the *B. pseudomallei* reference genome using Illumina HiSeq and PacBio RS II reads. Graph showing the base per base depth of sequencing coverage across the *B. pseudomallei* reference genome with **(A)** 143× Illumina HiSeq reads and **(B)** PacBio RS II reads (143×) using BBMap. Chromosome 1 (red) and chromosome 2 (blue). The regions of significantly higher coverage in both Illumina HiSeq datasets represent one ribosomal operon (spike 5) and multiple copies of mobile element proteins (spikes 1–4). PacBio dataset, on the other hand, revealed a more uniform coverage across the reference genome.

To investigate the distribution of reads (evenness of coverage) produced by the PacBio and Illumina platforms, BBMap was used to align a total of 114,845 subreads containing 1,131,636,843 bp from the PacBio dataset and 23,995,650 paired-end reads containing 6,278,193,333 bp from the Illumina dataset to the *B. pseudomallei* reference genome sequence. As the average depth of coverage of the *B. pseudomallei* genome represented by the PacBio data was only 143× whereas that of the Illumina reads was 869×, fractions of the Illumina reads equivalent to 143× genome coverage were randomly selected to allow fair comparison, and such fractions of reads as well as complete 869× Illumina reads were both subjected to analysis and alignment to the *B. pseudomallei* reference genome sequence. It was observed that both the PacBio (143×) and Illumina reads (both 143× and 869×) covered 100% of the *B. pseudomallei* reference genome sequence (chromosomes 1 and 2), with the PacBio dataset showing a more uniform coverage of the genome by the PacBio dataset (**Figures 3A,B** and **Supplementary Figure S1**). In the Illumina dataset, five regions (spike 1: 57,569–58,588 nt; spike 2: 214,733–216,022 nt; spike 3: 231,406–231,792 nt; spike 4: 337,351–338,241 nt, and spike 5: 1,636,599–1,641,851 nt) showed extraordinarily high coverage of reads (**Figure 3A** and **Supplementary Figure S1**). Detailed analysis of these regions revealed the presence of one ribosomal operon in the region of spike 5, while the rest of the regions (spikes 1–4) were repetitive regions due to the presence of four different types of mobile element proteins with each type having 4 to 10 copies across the whole genome (**Figure 3A** and **Supplementary Figure S1**). On the other hand, coverage of the PacBio data was more evenly distributed in these five regions (**Figure 3B**).

### Characteristics of *De Novo* Assemblies

**FIGURE 4:**
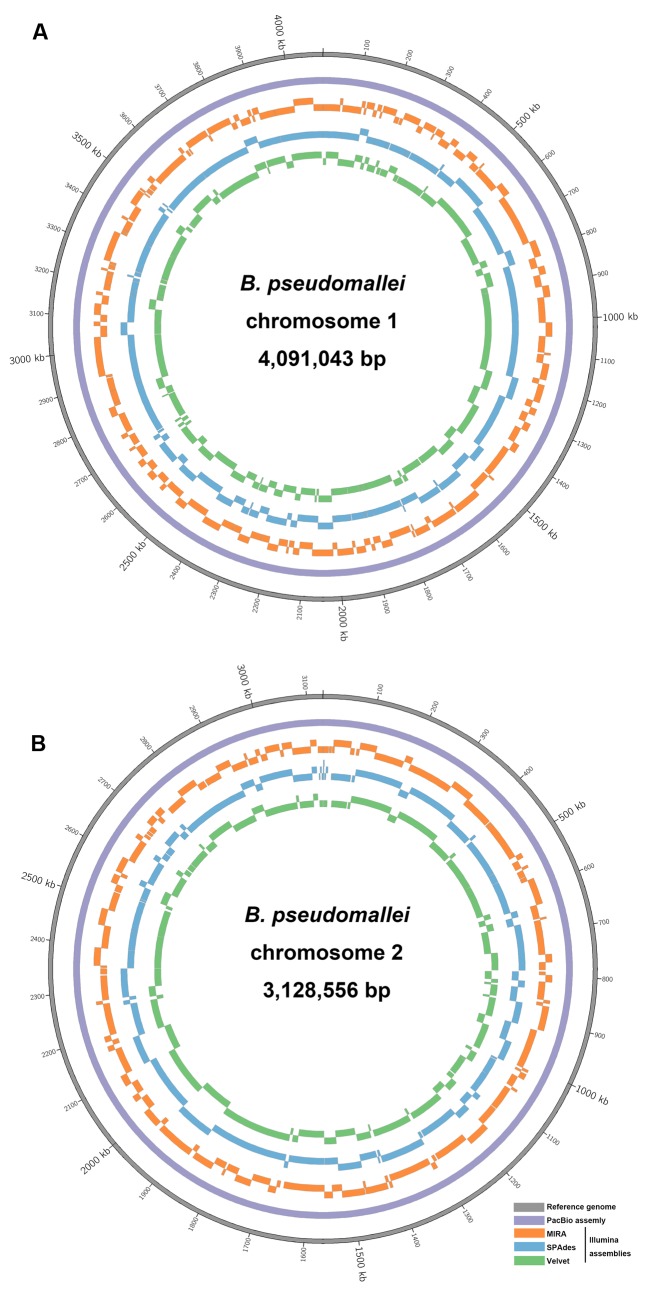
Circular representation of sequence comparison between the *B. pseudomallei* reference genome and other assemblies as labeled. **(A)** Chromosome I and **(B)** chromosome II. List of tracks (from inside to outside): Velvet contigs (green), SPAdes contigs (blue) and MIRA contigs (orange), PacBio contig (purple), and reference genome (gray). Each contig (only those >1,000 bp) was mapped to its respective positions on the reference genome by BLASR.

High-throughput sequencing can be used to identify differences in genome contents and arrangements, but the generation of accurate *de novo* assemblies is crucial for the analysis. An ideal case is that the final assembly generates a single accurate contig for each chromosome of *B. pseudomallei*, but this is difficult to achieve in many cases due to the presence of long repeat sequences and complex elements in the genome. In this study, after *de novo* assembly, heavily fragmented assemblies were obtained with the Illumina HiSeq data (MIRA: 366; SPAdes: 201, and Velvet: 288 contigs), with SPAdes producing the least fragmented assembly (**Table 2** and **Figure 4**). Similar results were obtained using complete 869× Illumina reads for *de novo* assembly with SPAdes and Velvet, respectively (Supplementary Table S2). On the other hand, only two large contigs were obtained with PacBio RSII data after *de novo* assembly. The output of these two contigs (4,091,945 and 3,130,290 bp) contained no gaps and did not have any ambiguous N bases.

**FIGURE 5:**
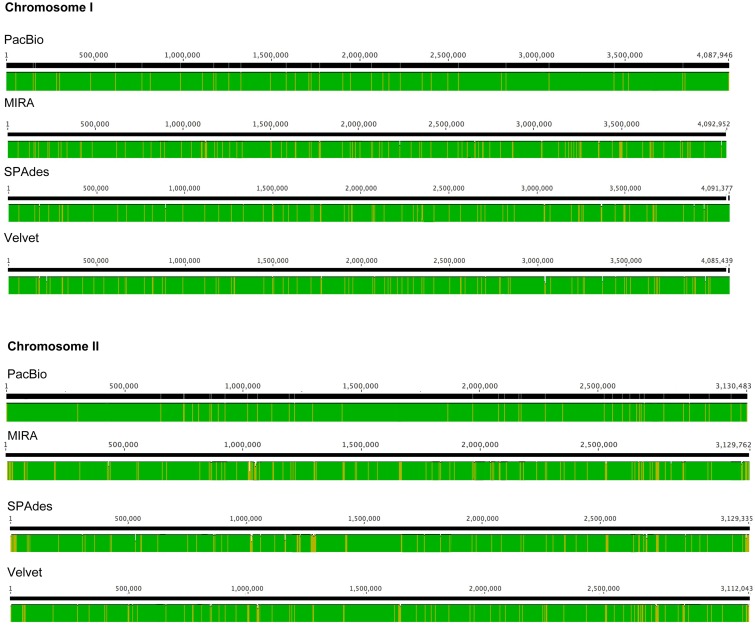
Base discordance between the *B. pseudomallei* reference genome and other assemblies as labeled. Color (Green: 100%; Yellow: 90–99% nucleotide identities) indicated level of similarity between each assembly and the reference genome.

**FIGURE 6:**
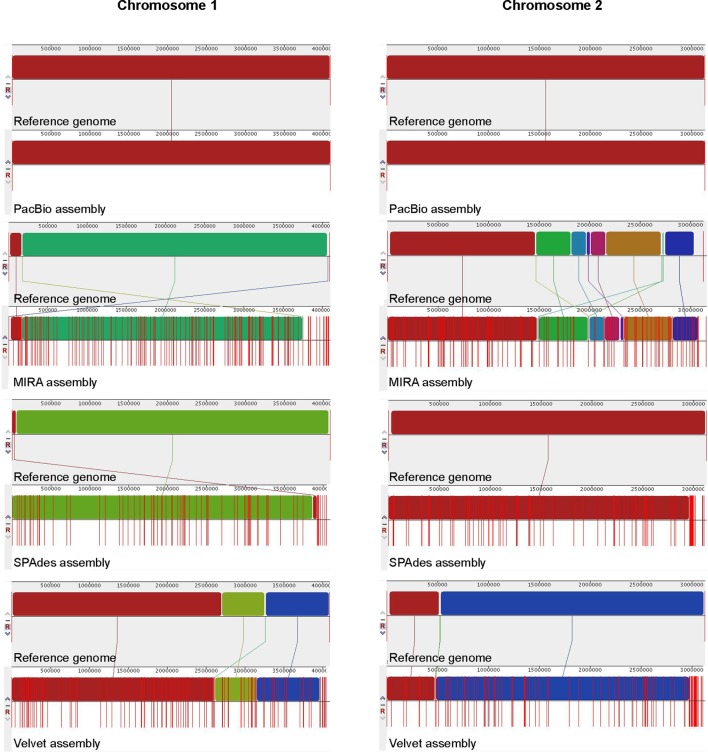
Whole genome alignment of *B. pseudomallei* genomes. The genome generated by each assembly was compared to the reference genome (chromosomes I and II) respectively, using Mauve. Locally collinear blocks represent homologous regions of sequence shared between the reference genome and genomes of other assemblies are represented with the same color and are connected by lines. Red vertical bars (lines) indicate contig boundaries.

The number of contigs that can be mapped unambiguously to the *B. pseudomallei* reference genome gives a measure of genome coverage. The two large contigs obtained from the PacBio RS II data alone aligned unambiguously to the reference genome (chromosomes I and II), sharing >99.9% nucleotide identities. No unmapped regions were observed for both chromosomes (**Figure 5**). Mauve analysis revealed a homologous region of sequence shared by the PacBio assembly and the reference genome forming a single contiguous collinear block, that suggested identical genome organization and the absence of mis-assemblies in the PacBio assembly (**Figure 6**). On the other hand, all Illumina assemblies showed disagreements with the reference genome, suggesting the presence of mis-assemblies in the Illumina assemblies (**Figure 6**). Moreover, contigs assembled from Illumina data shared only 92.2 to 100% and 92.0 to 100% nucleotide identities to chromosomes I and II reference sequences, respectively, (**Figure 5** and Supplementary Table S3). Base discordance between the Illumina assemblies and the reference genome were scattered across the whole genome (**Figure 5**). Among the Illumina contigs, 122 to 229 contigs could be mapped to chromosome I reference sequence, with 1,226 to 3,592 nt of the reference sequence uncovered. Another 151 to 219 contigs could be mapped to chromosome II reference sequence, with 1,305 to 3,730 nt uncovered. Of the 122 to 229 contigs, 72 to 83 contigs could be mapped to both of the chromosomes I and II reference sequences.

### Comparison of *De Novo* Assemblies on Resolving Repetitive Regions

**FIGURE 7:**
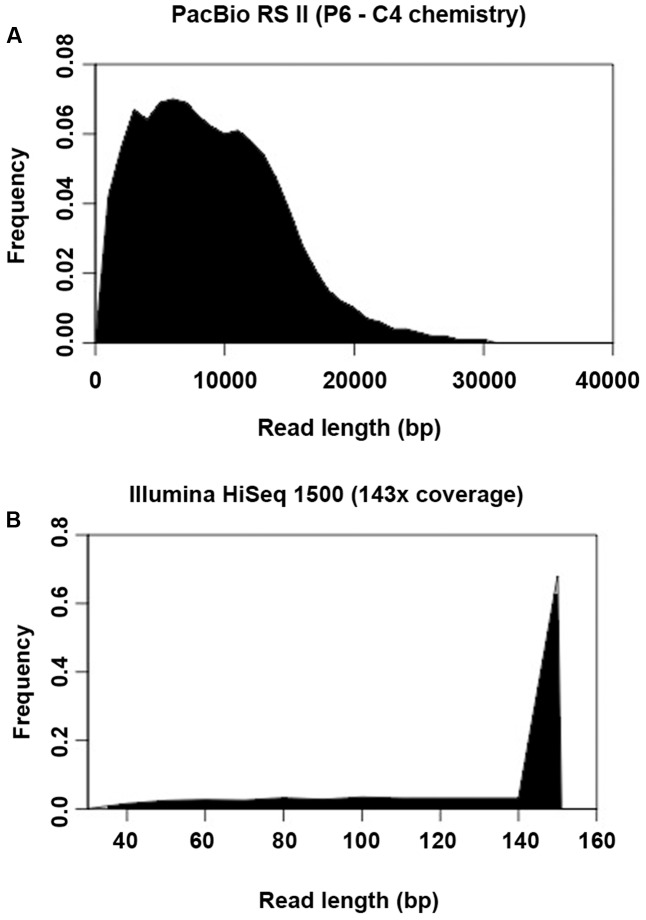
Histogram showing the read length distribution generated by **(A)** PacBio RS II and **(B)** Illumina HiSeq 1500.

Another important prerequisite for obtaining accurate genome assemblies is the resolution of repetitive regions. Sequences similar or identical to sequences elsewhere in the genome may cause mis-assemblies or fragmented assemblies especially if the sequence reads are too short to span the repeats with unique flanking sequences. This problem is often associated with next-generation sequencing (NGS) platforms which produce short read lengths, such as that generated by different models of Illumina platforms. In this study, there was a huge difference between the read length produced by PacBio (average read length was 8,711 bp) and Illumina HiSeq (average read length of 151 bp) (**Figures 7A,B**), leading to a difference in performance on resolving repetitive regions across the genome. For the PacBio assembly, 2,045 tandem repeats (4–984 bp) were found with copy numbers of 1.6–75.3, constituting around 2.3% of the genome. This result was highly comparable to that of the reference genome (**Table 3**). Although the number of tandem repeats detected in the assemblies of both platforms (PacBio 2,045; Illumina 2,042–2,088) was similar to the reference genome (2,052), the copy number of the repeats detected was much lower in the Illumina assemblies (up to 51.7 copies only) compared to the reference genome and PacBio assembly (up to 69.3 and 75.3 copies, respectively) (**Table 3**).

**Table 3:** Summary of number of repeats predicted by Tandem Repeats Finder.

Platform	Reference genome (hybrid assembly)	PacBio RS II	Illumina HiSeq 1500		
			
Assembler	SPAdes	SMRT analysis software suite	MIRA	SPAdes	Velvet
Number of repeat	2,052	2,045	2,088	2,042	2,053
Number of copy	1.8–69.3	1.8–75.3	1.8–51.7	1.8–36.9	1.8–35
Period size (bp)	4–954	4–954	4–954	4–954	4–834
Total length (bp)	159,618	162,531	160,870	159,741	152,134
Percentage of genome	2.3%	2.3%	2.2%	2.2%	2.1%


**FIGURE 8:**
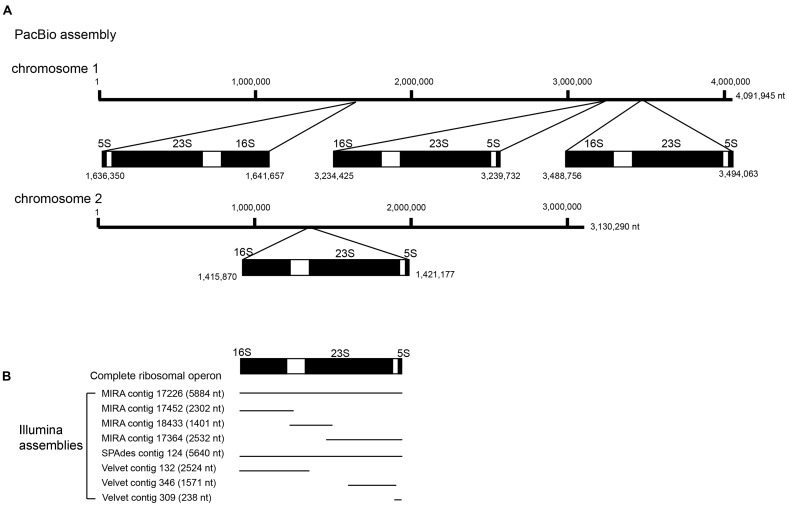
Schematic diagram of the ribosomal operons detected in different assemblies. **(A)** Location of the four complete copies of ribosomal operons in PacBio assembly was shown, with three copies distributed on chromosome 1 and one copy on chromosome 2, **(B)** among the Illumina assemblies, fragmented copies of ribosomal operons were detected in eight different contigs as labeled.

In addition to tandem repeats, the ribosomal operon represents another type of repeat that spans more than 5 kb in most bacteria. In this study, RNAmmer unambiguously predicted four complete copies of ribosomal operons (16S-23S-5S rRNAs) in the reference genome sequence. These ribosomal operons were distributed over both chromosomes, with three copies in chromosome 1 and one copy in chromosome 2. The organization and distribution of ribosomal operons was completely in concordant with those predicted by the assembly of the PacBio RS II data alone (**Figure 8**). This organization was also consistent with many other *B. pseudomallei* genomes available, such as *B. pseudomallei* strains K96243, 1710b, 668 and 1106a (Genbank accession numbers NC_006350/NC_006351, NC_007434/NC_007435, NC_009074/NC_009075, and NC_009076/NC_009078). In comparison, among the three assembly methods used to assemble the Illumina datasets (**Figure 8**), one complete copy of rRNA operon (16S-23S-5S) was found using the MIRA and SPAdes assemblies. In addition to this one complete copy of rRNA operon, one complete sequence of 16S rRNA, one complete sequence of 5S rRNA and a partial sequence of 23S rRNA were detected in different contigs of the MIRA assembly (**Figure 8**). As for the contigs assembled by Velvet, no complete rRNA operon (16S-23S-5S) could be recovered but a complete sequence of 16S rRNA, and partial sequence of 5S and 23S rRNAs were detected in three different contigs (**Figure 8**).

### Biological Consistency of the Assemblies

One of the most important applications of genome-sequencing is to generate credible new insights into the biology of the organism under investigation. *De novo* assemblies generated using data from each platform were assessed in their ability to report features of biological interest in the patient isolate. We used 3,909 core protein sequences, and 146 proteins sequences previously linked to the pathogenesis of *B. pseudomallei* as queries in translated BLAST searches of all assemblies obtained from the PacBio RS II and HiSeq platform. Using 80% coverage and 80% amino acid identity as threshold, BLAST results showed that the PacBio RS II assembly was able to capture the highest number of complete sequences of both core (*n* = 3,804) and virulence (*n* = 137) proteins, and the results were completely concordant to those using reference genome sequences (**Table 4**). Among the three assemblies using Illumina datasets, the SPAdes assembly performed the best, and was able to capture the highest number of core (*n* = 3,803) and virulence (*n* = 137) proteins, followed by the Velvet (3,802 core and 137 virulence proteins) and MIRA (3,787 core and 136 virulence proteins) assemblies (**Table 4**). Although the performance to recover these important proteins using the threshold value of 80% exhibited minimal differences, some of the protein alignments within this range were of different coverage (i.e., full-length vs. partial-length identical matches) and/or identities in different assemblies. Manual inspection of the BLAST results found that most of the differences were due to fragmented assemblies obtained from Illumina datasets. For example, query protein sequences were observed to align to the end of a contig at contig breaks or to different contigs. Furthermore, we investigated whether the assemblies from each platform could generate the correct MLST profile for this isolate, which was previously determined to be of sequence type (ST)-70 by conventional PCR and DNA sequencing using primers suggested by MLST website for typing of *B. pseudomallei*. The sequences of seven house-keeping genes were used as queries to search against the PacBio assembly, the three assemblies from Illumina HiSeq as well as the reference genome sequence using BLASTn and the results showed that all assemblies generated an identical MLST profile (**Table 4**). On the other hand, genome-wide analysis using the wgMLST approach showed that allelic profiles generated by each assembly were different to each other. Compared to the allelic profile generated by the reference genome sequence, only 11 mismatched nucleotides were detected in the PacBio assembly, while 55–101 mismatched nucleotides were found in the Illumina assemblies, indicating that the PacBio assembly outperformed the other Illumina assemblies (**Table 4**).

**Table 4:** Recovery of important *B. pseudomallei* proteins in different assemblies.

Platform	Reference genome (hybrid assembly)	PacBio RS II	Illumina HiSeq 1500		
			
Assembler	SPAdes	SMRT analysis software suite	MIRA	SPAdes	Velvet
Core proteins (*n* = 3,909)	3,804	3,804	3,787	3,803	3,802
Virulence factors (*n* = 146)	137	137	136	137	137
Actin-based motility (*n* = 1)	1	1	1	1	1
Adhesin (*n* = 13)	11	11	11	11	11
Antiphagocytosis (*n* = 25)	25	25	25	25	25
Invasion (*n* = 55)	53	53	53	53	53
Secretion systems (*n* = 52)	47	47	46		
MLST genes (*n* = 7)	7	7	7		
wgMLST genes^a^ (*n* = 5,689)	5,689	5,678	5,588	47 7 5,634	47 7 5,654


*^a^Total number of genes (3,394 in chromosome I and 2,295 in chromosome 2) present in all assemblies.*

## Discussion

In this study, we have shown that the PacBio RS II sequencing platform, using P6-C4 chemistry and *de novo* assembly, was able to completely sequence the genome of *B. pseudomallei* with the absence of gaps or major detectable errors and accurately identified the bacterium as a two-chromosome genome with four ribosomal operons. Illumina HiSeq 1500, on the other hand, generated a series of fragmented contigs with disagreements and mis-assemblies. The original PacBio RS sequencing platform (P1-C1) released in 2011 was able to generate maximum read lengths of ∼23,000 bases with an average read length of ∼2,500 bases. Since then, the company released improved versions of sequencing chemistries and in 2013, it released the P5-C3 chemistry which extends sequencing read lengths to an average of 8,500 bases. This platform was mostly used in combination with the Illumina platforms and/or 454, as a hybrid approach, to provide substantial improvement in the quality of genome assembly compared to using the second generation sequencing platforms alone. There are currently 79 complete genome sequences of *B. pseudomallei* deposited in NCBI, for which most of them have been assembled using the hybrid approach. However, in some cases, this strategy has been demonstrated to be inefficient in producing complete bacterial genomes, such as for two-chromosome bacterial species like *Burkholderia* species and *Rhodobacter sphaeroides* (Utturkar et al., 2014; Liao et al., 2015). Moreover, hybrid approach often requires careful optimization of assembly algorithms in order to enhance the continuity of draft assemblies (Liao et al., 2015). With gradual technical advancement and improved polymerase and chemistry (P6-C4), average read lengths of 10,000 to 15,000 bases, with the longest reads exceeding 40,000 bases, can be achieved with the present PacBio RS II sequencing platform. Such long reads have greatly enhanced the accuracy of genome assembly, particularly in regions with various repeats, such as ribosomal operons, mobile element proteins, direct and inverted repeats, and those with long homopolymer stretches. Recently, few studies have reported the complete genome sequencing of *Burkholderia* species using the PacBio RS II system with earlier sequencing chemistry or multiple SMRT cells (Belcaid et al., 2015; Bugrysheva et al., 2015; Elschner et al., 2017), suggesting the potential of using PacBio technology alone in resolving more complex genomes. In this study, the notoriously difficult-to-sequence *B. pseudomallei* genome was completed using PacBio RS II with just one SMRT cell, with the number of CDSs and their distributions in each subsystem (**Figure 1**), four ribosomal operons (**Figure 8**), the highest number of core and virulence proteins (coverage of query protein sequence and amino acid identity ≥80%), and MLST gene loci correctly assembled and recovered (**Table 4**). In fact, the two large contigs obtained from the PacBio assembly aligned unambiguously to the reference genome generated by a combination of the PacBio and Illumina sequences with subsequent manual validation using more than 100 PCR sequencing reactions (**Figure 4** and Supplementary Table S1). Mauve alignment confirmed that there were no mis-assemblies in the PacBio assembly (**Figure 6**). Consistent to previous studies, attempts to finish the genome by the hybrid approach using both PacBio and Illumina dataset were not successful in this study, with only 74 contigs formed. On the other hand, genome assembly by the non-hybrid approach using data generated by the PacBio RS II platform alone with the latest P6-C4 chemistry achieved complete closure of this two-chromosome *B. pseudomallei* genome without additional costly bench work and further sequencing, demonstrating its utility in the complete sequencing of bacterial genomes, particularly those that are well-known to be difficult-to-sequence.

In contrast to the PacBio RS II sequencing platform, assembly of the Illumina HiSeq 1500 sequences alone resulted in a draft genome with more than 200 contigs. Since the release of the *Haemophilus influenzae* genome in 1995 (Fleischmann et al., 1995), most bacterial genomes sequenced using Sanger sequencing in the late 1990s were sequenced and assembled to full completion. However, due to the high cost of completing a genome and the introduction of second generation sequencing platforms, most bacterial genomes sequenced in the late 2000s were only draft genomes, usually consisting of hundreds of individual contigs, with the exact number depending on the coverage of sequencing, the G+C content and complexity of the genome being sequenced. Although the current Illumina technology using MiSeq instrument (MiSeq PE300) can offer longer possible reads with sequence lengths up to 300 bp, fragmented assemblies are still observed, especially for bacterial genome with multiple chromosomes (Hsueh et al., 2015; Patil et al., 2017). In these draft genomes, the G+C content, gene content and pan-genome comparison of the bacteria can usually be analyzed in high accuracy, as demonstrated by the data generated by the Illumina HiSeq platform in the present study. It was noted though that the platform could not correctly assemble genomes with multiple replicons and regions with repeated sequences longer than the length of the sequenced reads nor could it determine the true copy number of repetitive elements. Although Illumina HiSeq sequencing can provide abundant coverage of a genome, the amplification biases as well as short read lengths of this technology often lead to fragmented and/or mis-assemblies, particularly in genomes with complex repeats. As demonstrated in this study, extraordinarily high coverage of Illumina reads were observed in several collapsed repeat regions, including regions containing varying copies of mobile element proteins and ribosomal operon (spikes 1–5; **Figure 3A**). Similar problems also extended to the copy number of tandem repeats and ribosomal operons with the copy number of tandem repeats detected in Illumina assemblies being much lower than that of the PacBio assembly (**Table 3**). Furthermore, in contrast to the PacBio assembly which resulted in four complete copies of rRNA operons, some of the predicted rRNAs in the three Illumina assemblies were only partial (**Figure 8**). We reasoned that Illumina sequencing was not able to resolve these repeat regions as their sequence reads were not long enough to span different kinds of repeats with unique flanking sequences. After *de novo* assembly, 201–366 contigs of >200 bp were generated using the Illumina HiSeq platform with three different assembly software (**Table 2**), with no indication that the *B. pseudomallei* genome consisted of two chromosomes, a unique phenomenon distinct from the usual single-chromosome genome in most bacterial species. Although the performance to recover core and virulence proteins was similar among the different assemblies using the threshold value of 80% in BLAST search (**Table 4**), detailed analysis of sequence alignments revealed that Illumina assemblies were only able to recover partial sequences in some of these important proteins. This is mainly due to genome mis-assembly or fragmented genomes in nature causing some of the protein sequences to fall into two or more different contigs. In fact, these problems were often observed in other draft bacterial genome sequences generated using second generation sequencing technologies (Kopf et al., 2014; Tushar et al., 2014; Xu et al., 2016).

In addition to its robustness, the generation of complete bacterial genomes using the PacBio RS II sequencing platform is cost-effective. Although the sequencing cost per Gb of using the Illumina platforms is lower than that of PacBio, high degree of multiplexity is required to make use of this advantage (**Table 5**). However, it would be impractical and complex in most cases to sequence hundreds or thousands of bacterial genomes in the same run. Furthermore, to completely sequence a bacterial genome using Sanger sequencing or the second generation sequencing platforms, the main bulk of the cost, labor and time is spent in the gap-filling phase. It has been estimated that when using these second generation sequencing platforms, around 95% of the money and time are spent in completing the last 1% of the bacterial genome. The extra labor cost and time spent to improve the draft genomes by additional sequencing for gap-filling or to determine the order and orientation of contigs are considerably high and negates any initial cost savings (**Table 5**). In contrast, although the cost per base is more expensive for the PacBio RS II platform compared to short-read sequencing technology, no additional manual work after *de novo* assembly is required and the benefit of obtaining an accurate number of individual replicons and an intact assembly of repetitive regions and mobile genetic elements justify the initial cost. In particular, these complex and repetitive elements have been shown to be relevant and important to evolution and diseases, providing good foundation for comparative and evolutionary genomic studies (Fraser et al., 2002; Gomez-Valero et al., 2014; Rhoads and Au, 2015; Bowden et al., 2016). Therefore, completing bacterial genomes should no longer be regarded as a luxury, but rather as a cost-effective necessity. With the above mentioned advantages of completing a bacterial genome as well as its ability to facilitate downstream transcriptomic, proteomic and metabolomic studies, bacterial genomes should be sequenced to completion if the facility is available. During the time of writing, the Pacific Biosciences company has released a new model, Sequel^TM^ System, to the market. The new model is based on the same technology as the PacBio RS II but can generate about seven times as many reads per SMRT cell. More importantly, the cost of this new instrument is approximately half of the previous platform. Further studies should be performed to evaluate the robustness of this new model in sequencing bigger and more complex genomes, such as those of fungi, protozoa and helminthes.

**Table 5:** Comparison of the PacBio RS II and Illumina HiSeq platforms used in this study^a^.

	PacBio RS II (P6-C4 chemistry)	Illumina HiSeq 1500 (rapid run mode)
Instrument price (US$)	$700,000	$690,000
Read length	8 to 15 kb	2 × 151 bp
Throughput per run	Up to 1 Gb	Up to 90 Gb
Instrument run time	4 h	40 h
Cost per Gb (US$)	$300	$55
Extra labor cost^b^	Nil	Yes
Extra time for completing the genome^b^	Nil	≥6 months


*^a^Data obtained from manufacturer, local distributor and 2016 NGS field guide at http://www.molecularecologist.com/*

*^b^Extra labor cost for at least 6 months for a research staff who will be responsible for designing primers and performing PCR and sequencing to fill the remaining gaps in the draft genome and to determine the unknown order and orientation of a collection of contigs. This extra cost does not include reagent and sequencing costs.*

### Data Availability

PacBio RS II data from this study has been submitted to the NCBI GenBank under accession numbers CP019042-CP019043 and Bioproject PRJNA342555. Raw data of PacBio RS II and Illumina HiSeq have been submitted to Sequence Read Archive (SRA) under accession numbers SRR5282539 and SRR5337839.

## Author Contributions

JT conceived of the study, designed the study, performed the laboratory work, contributed to the interpretation of results and wrote the manuscript. MY, EC, and LJ performed the laboratory work. CL participated in genome assembly. YH participated in bioinformatics analysis. HT, SW, PS gave advice on the bioinformatics analysis. SL conceived of the study, revised the manuscript and contributed reagents. PW conceived of the study, designed the study, contributed reagents and wrote the manuscript. All authors read and approved the manuscript.

## Conflict of Interest Statement

The authors declare that the research was conducted in the absence of any commercial or financial relationships that could be construed as a potential conflict of interest.

## Footnotes

1 https://www.bioinformatics.babraham.ac.uk/projects/trim_galore/

2 https://sourceforge.net/projects/bbmap/

3 http://wgmlstdb.imst.nsysu.edu.tw/

## Supplementary Material

**FIGURE S1 |** Base-per-base coverage of the *Burkholderia pseudomallei* reference genome using Illumina HiSeq reads. Graph showing the base per base depth of sequencing coverage across the *B. pseudomallei* reference genome with all Illumina HiSeq reads (869× coverage) using BBMap. Chromosome 1 (red) and chromosome 2 (blue). The regions of significantly higher coverage in both Illumina HiSeq datasets represent one ribosomal operon and multiple copies of mobile element proteins.
