# Supplementary figures and images for: PacBio But Not Illumina Technology Can Achieve Fast, Accurate and Complete Closure of the High GC, Complex Burkholderia pseudomallei Two-Chromosome Genome
Source: Front Microbiol. 2017 Aug 2;8:1448. doi: 10.3389/fmicb.2017.01448 (PMC5539568; doi:10.3389/fmicb.2017.01448)

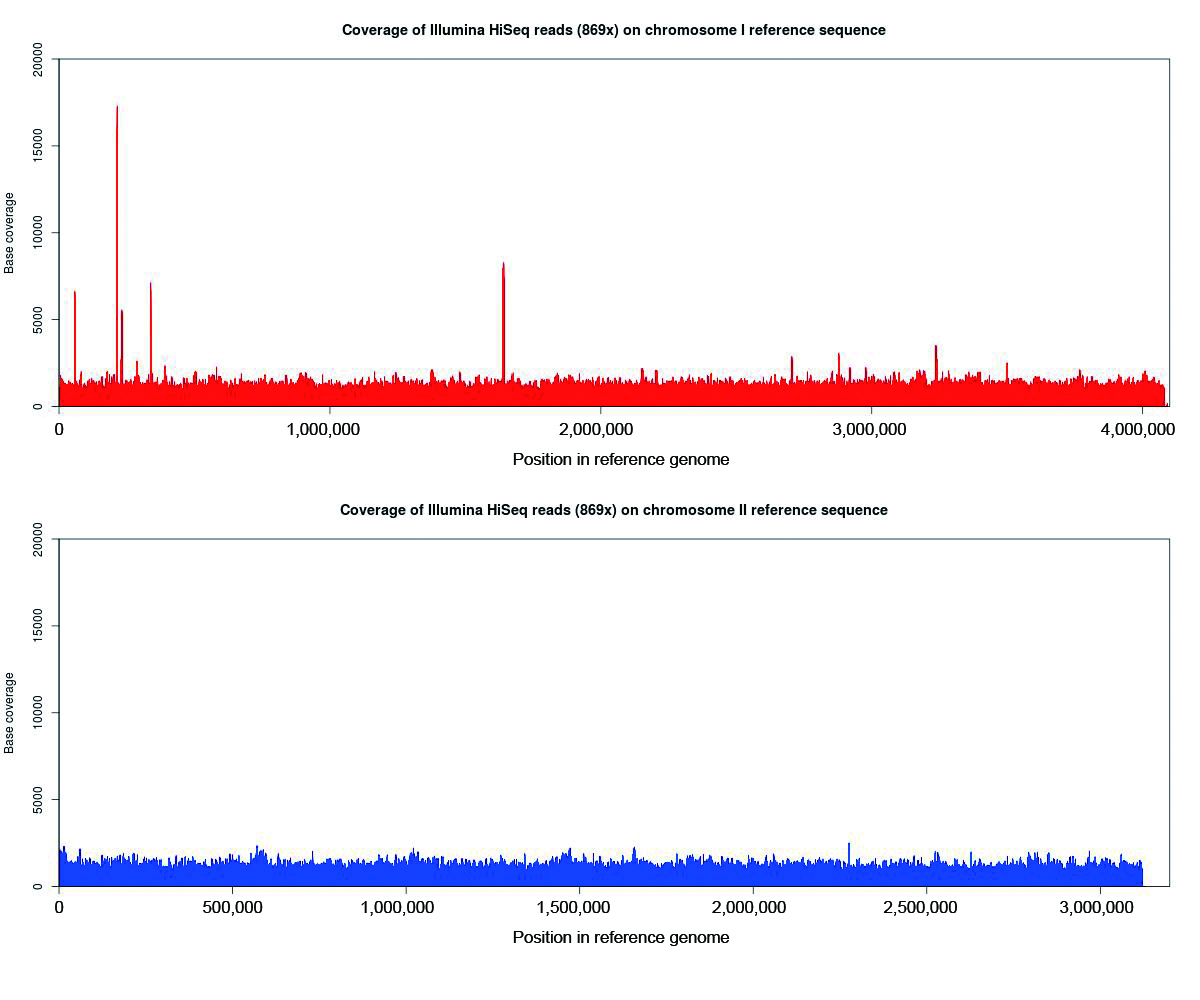

Supplement: Supplementary file 1 [file Image_1.TIF]
