# Supplementary material for: PacBio But Not Illumina Technology Can Achieve Fast, Accurate and Complete Closure of the High GC, Complex Burkholderia pseudomallei Two-Chromosome Genome
Source: Front Microbiol. 2017 Aug 2;8:1448. doi: 10.3389/fmicb.2017.01448 (PMC5539568; doi:10.3389/fmicb.2017.01448)
Supplement: Supplementary file 3 [file Table_2.DOC]

Supplementary Table S2. Genome characteristics for PacBio and Illumina platforms

| Platform |  | PacBio RS II (latest P6-C4 chemistry) |  | Illumina HiSeq | | |
| --- | --- | --- | --- | --- | --- | --- |
| Assembler |  | SMRT analysis software suite |  | MIRA | SPAdes^b^ | Velvet^c^ |
| Total no. of bases |  | 1,000,419,819 |  | 6,278,193,333 | 6,278,193,333 | 6,278,193,333 |
| No. of reads assembled |  | 114,845 |  | 4,296,615 | 47,991,300 | 47,991,300 |
| Average depth of coverage |  | 143× |  | 70×^a^ | 869× | 869× |
| Average read length (bp) |  | 8,711 |  | 151 | 131 | 131 |
| No. of contigs (>200bp) |  | 2 |  | 366 | 175 | 207 |
| Largest contigs (bp) |  | 4,091,945 |  | 152,181 | 673,378 | 439,538 |
| Assembled genome size (bp) |  | 7,222,235 |  | 7,261,126 | 7,104,327 | 7,109,717 |
| N50 |  | 4,091,945 |  | 45,496 | 118,739 | 91,084 |

^a^MIRA only allows *de novo* assembly with no more than 70× coverage of data

^b^Optimized k-mer values for SPAdes and Velvet were obtained respectively by KmerGenie (<http://kmergenie.bx.psu.edu/>) (Chikhi and Medvedev, 2014) and “VelvetOptimiser.pl”
